# Case report and literature review: PET/CT in the evaluation of response to treatment of liver metastasis from colorectal cancer with DEBIRI-TACE

**DOI:** 10.3389/fonc.2023.1015976

**Published:** 2023-03-02

**Authors:** Lan Jin, Wuyun Hu, Teng Li, Honghua Sun, Dongxu Kang, Longzhen Piao

**Affiliations:** Department of Oncology, The Affiliated Hospital of Yanbian University, Yanji, China

**Keywords:** colorectal liver metastases, DEBIRI-TACE, FDG PET/CT, irinotecan, prognosis, case report

## Abstract

**Background:**

Irinotecan-loaded drug-eluting beads transarterial chemoembolization (DEBIRI-TACE) is a safe and effective therapeutic option for unresectable colorectal liver metastases (CRLM). The evaluation of treatment response after DEBIRI-TACE is very important for assessing the patient’s condition. At present, the Response Evaluation Criteria in Solid Tumors (RECIST) with the tumor size obtained by CT and/or MRI and PET Response Criteria in Solid Tumors (PERCIST) based on fluorodeoxyglucose-positron emission tomography/computed tomography (FDG PET/CT) are used for evaluating the response to therapy of solid tumors; however, their value in the assessment of treatment response after DEBIRI-TACE remains unclear.

**Case presentation:**

A 52-year-old male with unresectable simultaneous CRLM was treated in the Affiliated Hospital of Yanbian University with DEBIRI-TACE combined with systemic chemotherapy and targeted therapy. Carcinoembryonic antigen levels decreased by 82.50% after 27 days of treatment. At 6 weeks post-surgery, FDG-PET/CT showed that the maximum standardized uptake value (SUVmax) of intrahepatic lesions was reduced to 62.14%. Abdominal MRI revealed that the sum of target lesion diameters was less than 30% that at baseline. PERCIST indicated partial metabolic response, whereas RECIST suggested stable disease.

**Conclusion:**

FDG PET/CT-based PERCIST may be accurate in determining treatment response and evaluating patient prognosis after DEBIRI-TACE in unresectable CRLM.

## 1 Introduction

Surgical resection is the best treatment option for colorectal cancer; however, approximately 15% to 30% of patients have liver metastasis at the time of diagnosis, with >80% being unresectable (1). Irinotecan-loaded drug-eluting beads transarterial chemoembolization (DEBIRI-TACE) has been increasingly used for treating unresectable colorectal liver metastases (CRLM) (2). In DEBIRI-TACE, irinotecan is slowly but continuously released by microspheres to exert its antitumor effects (1, 3). A phase III study (4) reported overall survival (OS) and progression-free survival (PFS) after DEBIRI-TACE of 7 and 3 months, respectively. These results were superior to those obtained with systemic intravenous chemotherapy (FOLFOX) and indicated that DEBIRI-TACE is a safe and effective treatment option for unresectable CRLM. Response Evaluation Criteria in Solid Tumors (RECIST) (**Table 1**) represent the most used tool for evaluating therapeutic effects on solid tumors, with tumor size obtained by CT and/or MRI serving as an evaluation index (5). PET Response Criteria in Solid Tumors (PERCIST) (**Table 1**) can help in the evaluation of treatment effects on solid tumors based on fluorodeoxyglucose-positron emission tomography/computed tomography (FDG PET/CT) (6). It can detect changes in the tumor microenvironment, quantitate the metabolism of glucose required for tumor growth, and evaluate treatment response in the early stage of therapy. In this study, we report a case of unresectable simultaneous CRLM after DEBIRI-TACE combined with systemic chemotherapy and targeted therapy and evaluate the therapeutic response using FDG PET/CT-based PERCIST and CT- or MRI-based RECIST.

**Table 1:** Comparison of RECIST and PERCIST.

RECIST		PERCIST	
CR	Disappearance of all target lesions. Any pathological lymph nodes (whether target or non target) must have reduction in short axis to <10mm.	CMR	Complete resolution of 18F-FDG uptake within the measurable target lesion(so that it is less than mean liver activity;so that it is at the level of surrounding background blood pool activity). Disappearance of all other lesions to background blood pool levels;no new suspicious 18F-FDG avid lesions.
PR	≥30% decrease in the sum of the longest diameters of the target lesions, taking as a reference the baseline sum of the diameters.	PMR	Reduction of a minimum of 30% in target measurable tumor 18F-FDG SUV, with absolute drop in SUV of at least 0.8 SUV units. No increase >30% of SUV or size in all other lesions. No new lesions
SD	Neither sufficient decrease to qualify for partial response nor sufficient increase to qualify for progressive disease.	SMR	No CMR, PMR, or PMD
PD	≥20% increase in the sum of the longest diameters of target lesions, taking as a reference the smallest sum on study. The sum must also demonstrate an absolute increase of at least 5mm. New lesions.	PMD	>30% increase in 18F-FDG SUV peak, with >0.8 SUV units increase in tumor SUV from the baseline scan in pattern typical of tumor and not of infection/ treatment effect; Visible increase in the extent of 18F-FDG tumor uptake; New 18F-FDG avid lesions typical of cancer and not related to treatment effect and/or infection

CR, complete remission; PR, partial remission; SD, stable disease; PD, progressive disease; CMR, complete metabolic response; PMR, partial metabolic response; PMD, progressive metabolic disease; SMD, stable metabolic disease.

## 2 Case description

**Figure 1:**
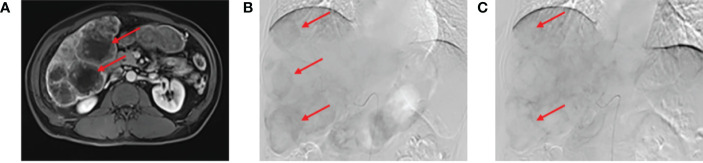
Abdominal magnetic resonance imaging (MRI) evaluation. **(A)** Abdominal MRI showing multiple intrahepatic lesions with a maximum diameter of approximately 5.7 cm. In the arterial phase before drug-eluting beads transarterial chemoembolization (DEB-TACE), multiple scattered abnormal vascular shadows could be seen in the right and left lobes of the liver **(B)**. One month after embolization, digital subtraction angiography (DSA) showed a significant reduction in abnormal vascular shadows in the tumor **(C)**.

In February 2020, a 52-year-old male was admitted to the Affiliated Hospital of Yanbian University presenting with persistent dull pain (10 minutes) in the liver that had lasted for 1 month. He had a history of diabetes for several years, with diet regulation. Physical examination revealed that the site of the pain was the upper abdomen. The carcinoembryonic antigen (CEA) level was 366.96 ng/L. Multiple intrahepatic space-occupying lesions, with a maximum diameter of approximately 5.7 cm, were found by MRI **(** **Figure 1A** **)**. To clarify the primary focus, FDG PET/CT was performed, resulting in the identification of transverse colon-occupying lesions and liver-occupying lesions, which were considered malignant. Further colonoscopy with biopsy showed villous tubular adenomas with high-grade intraepithelial neoplasia in the mucosal glands, suggestive of highly differentiated adenocarcinoma. The clinical diagnosis was “CRLM (cT3N0M1, stage IV)”. After multidisciplinary consultation and discussion, the treatment plan was established as DEBIRI-TACE combined with the CAPOX regimen and bevacizumab. DEBIRI-TACE was performed in February 2020. During the operation, multiple scattered, abnormal vascular shadows were observed in both the right and left lobes of the liver **(** **Figure 1B** **)**. CalliSpheres irinotecan-loaded (0.1 g) microspheres (Jiangsu Hengrui Jialisheng Biomedical Technology Co., Ltd; blue, 100–300 μm) were used for embolization. After the operation, the patient underwent systemic chemotherapy with the CAPOX regimen combined with 500 mg of bevacizumab (every 3 weeks). One month after embolization, digital subtraction angiography showed significantly smaller abnormal vascular shadows in the liver compared with the pre-treatment condition **(** **Figure 1C** **)**, and the CEA content had decreased to 64.21 ng/L. Tumor biomarker levels and intrahepatic tumor numbers were significantly reduced compared with before treatment. Considering that this treatment scheme was effective and safe, DEBIRI-TACE was performed again in March 2020. After the operation, systemic chemotherapy and targeted therapy were continued following the original scheme. The patient was discharged after the operation in good general condition and with no obvious discomfort.

**Figure 2:**
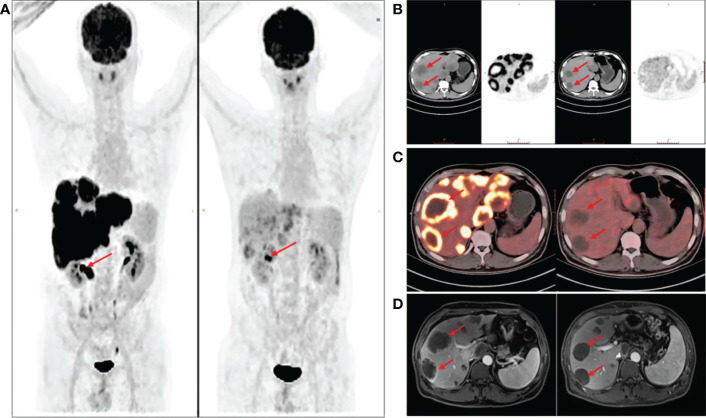
Drug-eluting beads transarterial chemoembolization (DEB-TACE), fluorodeoxyglucose-positron emission tomography/computed tomography (FDG PET/CT) assessment. Six weeks after the second DEB-TACE, FDG PET/CT **(A)** showed that the SUVmax of liver flexure and transverse colon lesions was decreased from 13.2 to 7.2. The SUVmax of some right lobe lesions was decreased from 14.0 to 5.3 **(B, C)** red arrow). Re-examination of contrast-enhanced abdominal MRI showed that the intrahepatic tumor volume was not significantly reduced, and the sum of target lesion diameters was less than 30% compared with the baseline level **(D)**.

In April 2020, FDG PET/CT **(** **Figure 2A** **)** showed a local thickening of the wall at the hepatic flexure and transverse colon, a narrowing of the lumen, increased FDG uptake (a decrease in the maximum standardized uptake value [SUVmax] from 13.2 to 7.2) in liver flexure and transverse colon lesions, slightly increased FDG uptake (a decrease in the SUVmax from 14.0 to 5.3) in right lobe lesions (**Figures 2B, C** **)**, and a decrease in hilar lymph node enlargement (a decrease in the SUVmax from 13.8 to 3.6). According to PERCIST classification (6), the treatment response was evaluated as partial metabolic response (PMR).

From April to June 2020, three cycles of systemic chemotherapy with the CAPOX regimen combined with bevacizumab were administered. In June 2020, contrast-enhanced abdominal MRI revealed multiple massive and abnormal nodular shadows in the liver with slightly enhanced edges. Some nodules and masses were slightly smaller compared with before treatment, and the sum of target lesion diameters was less than 30% of that of the baseline level **(** **Figure 2D** **)**. According to the RECIST standard, the treatment response was evaluated as stable disease (SD). The patient remains alive, with a PFS of 15 months and an OS of 27 months.

## 3 Discussion

The results of the present study indicated that FDG PET/CT-based PERCIST criteria may be accurate in identifying treatment responses for prognostic assessment after DEBIRI-TACE in unresectable CRLM.

The main goal of anticancer therapy is to improve the survival of patients and developing the best personalized treatment plan and timely imaging evaluation during treatment are vital for assessing treatment responses and prognosis. The widely used TACE allows the local delivery of high drug concentrations, blocks the blood supply to metastases, and improves treatment responses (7). Richter et al. (8) demonstrated that using DEBIRI-TACE, irinotecan can be continuously released at the tumor site, thus improving the treatment response in patients with liver metastasis by blocking the blood supply. Furthermore, DEBIRI-TACE is an effective palliative therapy for unresectable and chemoresistant CRLM, with a median survival of 13.3 to 25 months (9). Fiorentini et al. (4) and Martin et al. (10) showed that DEBIRI-TACE is better than systemic chemotherapy in terms of overall tumor response and PFS, and has no obvious side effects. In the current case, we administered DEBIRI-TACE combined with systemic chemotherapy and targeted therapy to quickly and effectively control the tumor load and primary liver lesions. Twenty-seven days after the first DEBIRI-TACE treatment, CEA was decreased by 82.50%. The intrahepatic tumor load was controlled after just two DEBIRI-TACE treatment cycles, thus displaying significant antitumor effects.

The evaluation of treatment response after DEBIRI-TACE is crucial for assessing the patient’s condition and guiding further treatment. In the case of our patient, FDG PET/CT re-examination 6 weeks after the operation showed that the SUVmax of intrahepatic lesions was decreased to 54.55%, and, based on PERCIST guidelines, the response evaluation was PMR. Meanwhile, MRI showed that tumor volume was not significantly reduced, the sum of target lesion diameters was less than 30% of that at baseline, and, according to the RECIST standard, the treatment response was evaluated as SD. The consistency of tumor response between PERCIST and RECIST was poor. RECIST relies only on tumor morphology and does not consider tumor necrosis. Additionally, its use may be limited for tumors with blurred contours or due to the presence of cystic lesions or scar tissue (11, 12). Interventional and targeted therapies are increasingly being employed, which may cause tumor necrosis without a substantial and concomitant change in tumor volume. Consequently, RECIST may be misleading in evaluating responses to these treatments (13).

The modified RECIST (mRECIST) is currently the most commonly used tool for evaluating tumor response after DEBIRI-TACE in CRLM (14–16). However, efficacy evaluation by the mRECIST standard is still limited to the measurement of changes in tumor volume through the functional imaging of viable tumors. Neither RECIST nor mRECIST accurately reflects the activity of residual tumors. Using MRI at the early stage could result in the underestimation of the efficacy of DEBIRI-TACE, misinforming the follow-up treatment and ultimately affecting patient prognosis and survival.

Changes in tumor size may lag behind the metabolic response for weeks or even months (17). FDG PET/CT imaging can evaluate the alterations in biological metabolic activity in the tumor during anticancer treatment. Consequently, this imaging modality can also be used for assessing both tumor activity and residual tumor activity, and may accurately identify disease progression and disease stability *via* PERCIST after DEBIRI-TACE in patients with CRLM.

Relatively few studies have directly compared the prognostic values of PERCIST and RECIST. In 44 patients with non-small cell lung cancer administered chemotherapy without surgery, PERCIST had good consistency with RECIST, although PERCIST was more sensitive in assessing complete remission and progression (11). In another study assessing 35 patients with non-small cell lung cancer after chemotherapy, the PERCIST standard showed a difference in PFS between patients with PMR and those with stable metabolic disease, whereas no significant difference in PFS was detected between these groups when RECIST was used. This suggested that PERCIST may be an important predictor of prognosis (18). In the era of precision medicine, PERCIST provides a more accurate treatment response evaluation *via* non-invasive imaging and may be more suitable than RECIST for evaluating tumor response to anticancer therapy based on the tumor’s biological and metabolic activities; however, further investigation is required to confirm this possibility.

FOLFOX or CAPOX in combination with bevacizumab or cetuximab remains the recommended first-line therapy in the National Comprehensive Cancer Network guidelines for patients with colorectal cancer (19). Comparative data are limited to DEBIRI-TACE in a phase III study involving previously treated patients showing a benefit versus systemic chemotherapy (4). The various guidelines do not provide a clear indication regarding the recommendation of DEBIRI-TACE as a first-line treatment for patients with CRLM. Only the 2016 ESMO guidelines suggest DEBIRI-TACE as a treatment option for these patients after the failure of first-line chemotherapy (20). However, patients who failed first-/second-line chemotherapy, especially those with irinotecan resistance, did not achieve satisfactory results even if treated with DEBIRI-TACE. In this study, the patient had an ECOG score of 0 and a large liver tumor load that could not be effectively controlled by chemotherapy combined with targeted therapy alone. We used DEBIRI-TACE combined with systemic therapy as a first-line therapy, which can rapidly block the blood supply to tumors and induce tumor cell necrosis. Treatment efficacy was evaluated using FDG PET/CT before and after treatment, and the SUVmax of intrahepatic lesions was decreased to 62.14%, indicative of significant efficacy. The patient experienced abdominal pain after DEBIRI-TACE treatment, but it was tolerated. Moreover, there was no treatment-related liver injury, suggestive of a good safety profile.

This case report demonstrated that the early application of FDG PET/CT may be the best choice for evaluating the postoperative response to DEBIRI-TACE in patients with CRLM. Additionally, the PERCIST standard may be more accurate than RECIST in judging complete remission and disease progression. Further prospective studies are needed to confirm these findings. A phase III clinical investigation is needed to validate the effectiveness of DEBIRI-TACE as a first-line treatment for CRLM and create a foundation for clinical research. Notably, however, even though the assessment of disease with FDG PET/CT before and after DEBIRI-TACE is expensive and may represent a financial burden for patients, it is not included in medical insurance in China. As a next step, we aim to use the biological features of left- and right-sided colorectal cancer, MSI/MMR status, and RAS/BRAF mutation status to compare the effectiveness of DEBIRI-TACE in CRLM.

## Data availability statement

The original contributions presented in the study are included in the article/supplementary material. Further inquiries can be directed to the corresponding author/s.

## Ethics statement

The studies involving human participants were reviewed and approved by the ethics committee of Yanbian University Affiliated Hospital. The patients/participants provided their written informed consent to participate in this study.

## Author contributions

LJ and WH wrote the manuscript. DK revised the manuscript. LP conceived the study. TL and HS performed the literature search. All authors contributed to the article and approved the submitted version.
